# Recent Advancements in the Relationship Between the Autonomic Nervous System and the Pancreas, Encompassing the Regulation of Regeneration and Apoptosis

**DOI:** 10.3390/cells14171371

**Published:** 2025-09-02

**Authors:** Takayoshi Kiba

**Affiliations:** Department of Medical Technology, Faculty of Life Sciences, Okayama University of Science, Okayama 700-0005, Japan; takkiba@hotmail.com

**Keywords:** advancements, apoptosis, autonomic nervous system, pancreas, regeneration

## Abstract

A previous review by the author reported relationships between the autonomic nervous system and the pancreas, including regulation of regeneration and apoptosis. This review documents the key clinical and laboratory features that have either been discovered since the previous update (published August 2004) or were characterized earlier but have since been confirmed or expanded in subsequent studies. These advancements regarding regulation of insulin secretion, pancreatic regeneration, apoptosis and carcinogenesis, and gene expression and growth factors provide a deeper understanding of how the autonomic nervous system interacts with the pancreas, offering potential avenues for therapeutic interventions in pancreatic diseases.

## 1. Introduction

Recent research has uncovered various genes and growth factors that become active post-birth and play a role in the differentiation, growth, and regeneration of the pancreas. The autonomic nervous system affects these factors, highlighting its importance in maintaining pancreatic health. The autonomic nervous system plays a crucial role in managing pancreatic regeneration. Research indicates that neural activity can affect the growth of pancreatic cells and the creation of new cells via replication and neogenesis. These findings enhance our understanding of the relationship between the autonomic nervous system and the pancreas, opening up possible therapeutic options for pancreatic disorders. Additionally, this information is vital for gaining insights into the development of diabetes and pancreatic cancer, as well as for formulating better treatment approaches for these conditions.

A previous review by the author reported relationships between the autonomic nervous system and the pancreas, including regulation of regeneration and apoptosis [1]. This review documents the key clinical and laboratory features that have either been discovered since the previous update (published August 2004) or were characterized earlier but have since been confirmed or expanded in subsequent studies.

## 2. Gene Expression of the Pancreas

**Figure 1:**
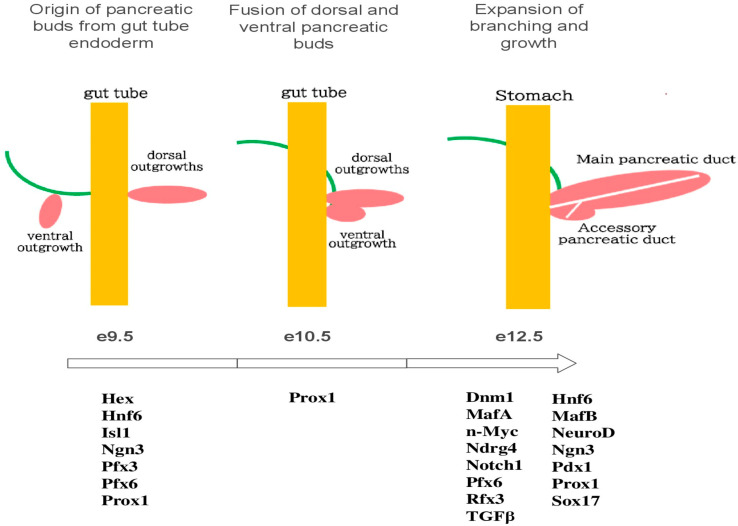
Illustrates the primary gene expressions involved in the embryonic development of the pancreas of mice.

Figure 1 illustrates the main gene expressions involved in the embryonic development of the pancreas. While certain growth signals have clearly defined roles in differentiation and proliferation, the specific functions of many growth signals remain unclear [2]. Besides the usual cell cycle regulators, some local signals have been identified that influence β-cell differentiation and characteristics.

In mice, the pancreas begins to develop around e9.5 from dorsal and ventral outgrowths of the gut tube endoderm [3]. These pancreatic buds grow into the surrounding visceral mesoderm. Soon after e10.5, the gut tube rotates, leading to the merging of the dorsal and ventral pancreatic buds into a single organ [3]. The pancreatic epithelium then starts to branch out, forming short, finger-like lobules that extend into the mesoderm. From e12.5 until birth, this branching continues, resulting in a three-dimensional organ that undergoes significant growth throughout gestation [3]. During this period, the pancreatic epithelium experiences various dynamic cellular transformations, developing into a tree-like, tubular epithelial structure. Along the main trunk of this developing structure endocrine progenitor cells detach as individual cells from the endodermal epithelium. These progenitor cells migrate and cluster together to form small islet-like groups, which gradually merge and expand into larger endocrine aggregates [3]. Significant cell proliferation and differentiation lead to the formation of both the exocrine pancreas (comprising acinar tissue and ductal epithelium) and the endocrine pancreas (which includes glucagon-secreting α cells, insulin-secreting β cells, somatostatin-secreting δ cells, pancreatic polypeptide-secreting PP cells) as well as ghrelin-secreting ε cells [4]. This process establishes the digestive exocrine pancreas and functional islets that help regulate glucose homeostasis. Additionally, a crucial factor in determining β-cell fate is the Notch signaling pathway. It has been proposed that the Notch 1 receptor, which is present in various lineages within the islets, can inhibit the differentiation of both exocrine and endocrine cells, with the differentiation of endocrine cells being vital for the development of insulin-producing and insulin-sensing capabilities.

The pancreatic epithelium reacts in a cell-autonomous way to specific levels of Ngn3 (neurogenin 3), which influences the number of cells directed towards the endocrine pathway [5]. Ngn3 is a basic helix-loop-helix (bHLH) transcription factor essential for the development of endocrine cells in the islets [3]. The expression of both *Ngn3* mRNA and protein occurs in two separate temporal waves: the first wave takes place early, around e8.5 to e11.0, and the second begins around e12.0. This biphasic expression pattern aligns with two distinct phases of embryonic endocrine differentiation. The terminal differentiation of β cells occurs normally in cells that lose hepatocyte nuclear factor 6 (Hnf6) after the *Ngn3* promoter is activated, as Hnf6 is specifically involved in the early stages of endocrine specification [6]. Transforming growth factor beta (TGFβ) signaling regulates the quantity and differentiation of β cells by enhancing their differentiation [7]. NeuroD1 (neurogenic differentiation 1) is another vital transcription factor that facilitates islet cell differentiation [8] and is believed to convert progenitor cells or cells from other organs into β cells [9]. The abnormal expression of pancreatic and duodenal homeobox 1 (Pdx1) in the endocrine pancreas leads to β-cell inactivity [10]. NeuroD1, Pdx1, and MafA bind directly to the insulin gene promoter and are crucial transcription factors for the differentiation and maintenance of pancreatic β cells [11]. NeuroD1, Pdx1, and MafA significantly enhance insulin production in various non-β cells, making it an effective method for generating insulin-producing surrogate β cells [11]. While other related transcription factors are produced in the pancreas, such as paired box gene 4 (Pax4)/paired box gene 6 (Pax6), NK homeobox 6.1 (Nkx6.1)/NK homeobox 6.2 (Nkx6.2), and forkhead box A1 (FoxA1)/forkhead box A2 (FoxA2), none appear to influence islet cell function as specifically and effectively as MafA and MafB [12]. MafB seems to be a stronger regulator of β-cell development compared to MafA [12]. Additionally, MafB activates genes related to mature endocrine functions, including those involved in glucose sensing, vesicle maturation, and insulin secretion. MafB is more prominent in the early stages of pancreatic development, but as the endocrine pancreas matures into functional islets, MafA takes on a more significant role. MafA regulates many genes initially controlled by MafB in developing β cells [12]. During pancreatic development, the first insulin-producing cells express MafB and later transition to MafA following the induction of Nkx6.1 and Pdx1, similar to mature β cells [7].

Prospero homeobox 1 (Prox1) is found in almost all pancreatic progenitor cells during early development [13]. It begins to be expressed in endodermal cells at e7.5 and facilitates the development of endocrine cells as various pancreatic lineages commit [7]. The expression of islet1 (Isl1) in the dorsal mesenchyme is essential for its maintenance and indirectly supports the differentiation of the exocrine pancreas, while Isl1 in pancreatic progenitor cells is crucial for the differentiation of the endocrine pancreas [7]. Hematopoietically expressed homeobox (Hex) is not necessary for determining the ventral pancreatic fate, but it is important for positioning pancreatic progenitor cells at the forefront of the ventral embryonic endoderm, allowing them to avoid the effects of mesenchymal inhibitors [7]. In the development of the vertebrate pancreas, pancreas-specific transcription factor 1a (Ptf1a) is believed to play a crucial role in determining pancreatic identity, making decisions between exocrine and endocrine cell types, and preserving the identity of acinar cells, as well as having functions related to neurodevelopment [14]. Genetic cell tracing studies have shown that Ptf1a is a reliable pancreatic marker, even more so than Pdx1, which is expressed earlier but is not limited to the pancreas [7].

Mammals have seven Rfx (regulatory factor X; Rfx1–Rfx7) proteins [15]. These Rfx transcription factors are involved in the development of islets [16]. Specifically, Rfx3, similar to Rfx6, plays a role in the formation of pancreatic endocrine cells, including β cells. Rfx3 is essential for the differentiation and proper functioning of mature β cells [16]. Rfx6 is crucial for the development of pancreatic endocrine cells [17]. In mice, Rfx6 is initially expressed in the definitive endoderm during early development, then becomes localized to the gut and pancreatic bud, is reactivated in endocrine progenitor cells, and is ultimately confined to islets in adult mice [17]. Additionally, Rfx6 is found in human pancreatic tissue, showing a similar expression pattern to that observed in adult mice [17]. Rfx transcription factors are vital for the development of the pancreas and the maintenance of functional islets.

In the pancreas of neonatal rats, n-myc downstream-regulated gene 4 (Ndrg4) was detected in pancreatic duct cells and developing acinar tissue, but not in the endocrine cells of the islets [18]. The presence of both n-myc and Ndrg4 in pancreatic duct cells indicates similarities in the differentiation pathways of cells in the pancreas and the central nervous system. Hnf6 is crucial for the development of both ductal and endocrine tissues and likely operates upstream of the transcription factor Prox1, potentially influencing duct morphogenesis through Prox1 [6]. Overexpressing Sry-box transcription factor 17 (Sox17) in the pancreas at e12.5, a time when it is not typically expressed, is enough to drive the ductal fate at the expense of endocrine cells [19]. This implies that Sox17 plays a key role in initiating a transcriptional program that leads to ductal differentiation. Additionally, genomic hypomethylation due to impaired DNA (cytosine-5)-methyltransferase 1 (Dnmt1) activity is linked to an enhanced ability to generate de novo β cells following ablation [20]. The increased regeneration of β cells in *Dnmt1*-deficient zebrafish may stem from the reprogramming of fully differentiated pancreatic cells, the promotion of β-cell formation from multipotent progenitors, or simply an enhanced potential for endocrine cell differentiation in the absence of exocrine tissue [20].

## 3. Autonomic Nerve Innervation and Pancreatic Function and Regeneration 

**Figure 2:**
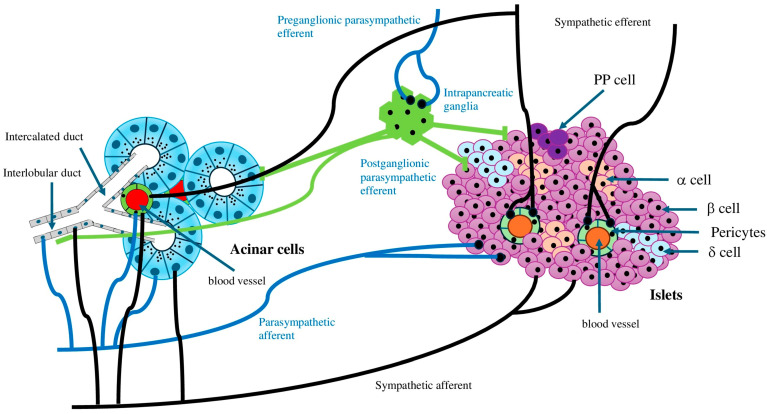
Depicts a diagram indicating that the islets and acinar cells are connected to the autonomic nervous system through both afferent and efferent signaling, which serves as the main regulatory pathway.

Research involving rodents indicates that the fetal stage is crucial for the development of pancreatic β-cell mass. During this time, islet β cells begin to proliferate in response to growth factors, hormones, and likely important signals from the autonomic nervous system [1]. The autonomic nervous system is vital for maintaining homeostasis and regulates the involuntary functions of most peripheral organ systems [21]. In various biological systems, such as the heart, eye pupil, digestive tract, glucose metabolism, and exocrine glands, the sympathetic nervous system is either opposed or complemented by the parasympathetic nervous system [1]. The vagus nerve and sympathetic nerve can be further divided into afferent and efferent nerve fibers, respectively [22]. The islets are heavily innervated [23], involving both the central and autonomic nervous systems with afferent and efferent signaling, with the vagus nerve serving as the primary regulatory pathway [24] (Figure 2). The pancreas has a rich supply of preganglionic vagal neurons [25]. Glucose-sensing cells located in various anatomical regions—the mouth, gut, hepatoportal vein area, brainstem, and hypothalamus—are interconnected through nervous pathways to ultimately regulate the sympathetic and parasympathetic innervation of the endocrine pancreas. The parasympathetic nervous system (vagus nerve) originates from the central nervous system and extends into the islets, facilitating insulin secretion from β cells and lowering hyperglycemia [1]. Conversely, the sympathetic nervous system arises from the paravertebral sympathetic ganglion chain and promotes glucagon secretion from α cells, resulting in elevated blood glucose levels [1]. Nerves that innervate the islets include peptidergic, cholinergic, adrenergic, and GABAergic fibers. Some of these nerves make direct synapses with endocrine cells, affecting their function [26]. Sympathetic innervation is significant in both the normal and abnormal functioning of the endocrine pancreas; noradrenaline and adrenaline inhibit insulin secretion while stimulating glucagon secretion [27]. Autonomic nerves synapse onto clusters of neurons in intrapancreatic ganglia, which are distributed throughout the pancreas in various animals, including mice, rats, cats, rabbits, and guinea pigs [28]. Similar to the enteric nervous system, an intrapancreatic nervous system develops, allowing the pancreas some degree of independence from the central nervous system and the gut. The pancreatic ganglia serve as integration centers for both exocrine and endocrine secretion. The fully developed pancreas is innervated by vagal preganglionic, sympathetic postganglionic, sensory, and enteric fibers (Figure 2). The pancreas’s autonomic nervous system interacts with ganglionic structures that are randomly distributed throughout the pancreatic tissue, representing the intrinsic neural component of the pancreatic nerve supply [1]. Neurons and nerve fibers form complexes with endocrine and epithelial cells in ducts early in the second trimester, including pain fibers from parasympathetic nerves [29]. Interestingly, the pancreas has the highest concentration of nerve innervation at its head, which diminishes towards the tail [30]. The sympathetic nervous system appears to develop during the early stages of fetal growth and may influence the development of the endocrine pancreas [29]. The results from experimental studies also show that the sympathetic nervous system plays a role in how endocrine cells develop and form islets during pancreas growth [29]. Recent reports indicate that the pancreas in humans forms nerve connections, including the development of nerve networks around blood vessels and within nerve groups, mainly during the prenatal period. However, some nerve connections, such as those to the capillaries in the islets, might happen after birth. The way sympathetic nerve endings interact with endocrine cells in the developing pancreas seems important for the proper development of the endocrine part of the pancreas in humans [29]. Additionally, evidence suggests that sympathetic activity may inhibit blood flow, leading to reduced exocrine secretion, indicating an indirect effect [31] (Figure 2). Conversely, parasympathetic innervation plays a significant role in exocrine function, primarily through vagal nerve activity, particularly during the cephalic phase [32]. Additionally, the gastric, intestinal, and nutrient absorption phases seem to involve both direct and indirect mechanisms (via the vagal nerve) for the secretion of pancreatic enzymes [33]. Studies on rodents showed that the parasympathetic afferent fibers come from blood vessels, ducts, acini, islets, and ganglia inside the pancreas. These fibers end in the space around small blood vessels but do not form connections with other nerve cells [22]. In humans, these nerve fibers are not as common in the islets compared to rodents [34]. These afferent neurons also make chemicals such as substance P and CGRP, which help control inflammation and the body’s response to mechanical pressure [35]. Like the parasympathetic nerves, sympathetic afferent nerves send signals from blood vessels, acini, islets, and ganglia inside the pancreas through the space around blood vessels. These nerves have TRPV1 channels and make substance P, CGRP, and NPY, which help them detect both chemical and mechanical signals [35].

Sympathetic neural cell bodies are found in the superior mesenteric and celiac ganglia, forming part of the splanchnic nerve, while parasympathetic innervation comes from the vagus nerve [36]. When blood glucose levels drop below normal, the autonomic nervous system is quickly activated to initiate a counterregulatory response aimed at restoring normal glucose levels. This response involves increased glucagon secretion, catecholamine release from the adrenal glands, and reduced insulin secretion [37]. The autonomic nervous system and the sympathoadrenal axis regulate these responses. Both sympathetic and parasympathetic systems stimulate glucagon secretion by directly activating α cells. The sympathetic system inhibits insulin secretion through α2AR-mediated pathways, while adrenaline release from the adrenal glands promotes glucagon secretion via β2AR activation and suppresses insulin secretion through α2AR in β cells. Additionally, the area of sympathetic fibers in diabetic islets from db/db mice was significantly larger compared to non-diabetic db/+ controls and obese non-diabetic ob/ob mice, which aligns with the observed link between increased noradrenergic fiber content and reduced insulin secretion in diabetes [38]. Another report supports the fact that adrenergic innervation has an inhibitory effect on insulin secretion, as it has been shown that β cells possess more α2ARs (which inhibit insulin secretion) than α1ARs and β2ARs (which promote insulin secretion) [39]. The hypothalamus and brainstem contain central glucose detection sites that activate the autonomic nervous system. Research involving lesions, pharmacological interventions, and genetic studies has identified these central sites responsible for counterregulation during hypoglycemia [40]. A loss of sympathetic input to islet cells leads to a diminished glucagon response and can trigger diabetes onset [41]. Therefore, spinal cord injuries that disrupt sympathetic regulation can affect glucose homeostasis. The autonomic innervation of the endocrine pancreas also influences the proliferation of pancreatic β cells, affecting islet mass in pathological conditions [1]. Additionally, the sympathetic innervation of islets is affected in animal models that exhibit insulin resistance and type 2 diabetes [42]. The parasympathetic nervous system promotes insulin secretion during feeding, while the sympathetic system inhibits it during stress. In type 2 diabetes, pancreatic neuronal activity is compromised [43]. The cephalic phase of insulin secretion is absent in type 2 diabetes, and glucagon secretion is not suppressed after eating. This indicates that the nervous system’s ability to suppress glucagon secretion in a manner independent of insulin during the fed state in healthy individuals is lacking in type 2 diabetes. It has been established that glucose-sensing cells in the central nervous system respond to changes in plasma glucose levels, and these neurons are thought to regulate the sympathetic and parasympathetic branches of the autonomic nervous system, which affect insulin and glucagon secretion [43]. The activation of the sympathetic and sympathoadrenal responses relies on hypoglycemia detection at various sites. Catecholamine release triggered by insulin-induced hypoglycemia can be inhibited by superior mesenteric ganglionectomy or capsaicin application to the hepatoportal vein, but not by vagal nerve transection [44]. This indicates a direct role of these peripheral glucose sensors in the autonomic nervous system’s regulation of α-cell secretion. For instance, the sympathetic control of insulin release is well understood, with adrenaline inhibiting insulin and promoting glucagon secretion [45]. Sympathetic innervation of the islets plays a role in the glucagon response following insulin-induced hypoglycemia [46]. It is important to highlight that in patients with type 1 diabetes, the sympathetic regulation of the glucagon response is diminished early on [47], which may be linked to the early onset of sympathetic islet neuropathy observed in BB rats [48]. Recent studies have shown that sympathetic innervation of the islets increases in experimental diabetic mice [49] and that the secretion of insulin relies on the tone of the autonomic nervous system [50].

In the developing pancreas of mice, neural crest cells and their derivatives are found near epithelial cells and endocrine β and α cells [51]. The innervation of islets in rodents begins during the early postnatal period. In mice, sympathetic and sensory nerve fibers innervate the islets between postnatal days 0 and 15, closely linked to the maturation of islets during this time [52]. Similar findings regarding the development of sympathetic innervation in islets during the first 20 postnatal days have been reported in rats [53]. It is believed that the balance between parasympathetic and sympathetic innervation is essential for the pancreas to function properly. In contrast, the autonomic innervation of the human pancreas develops significantly during the prenatal period and exhibits distinct growth patterns [54]. Large nerve bundles and clusters of poorly differentiated neurons can be found in the human fetal pancreas from early stages (8 weeks post-conception) [29]. The number of autonomic nerves increases significantly as the pancreas develops, with two notable peaks in growth (at 14 and 20 weeks of gestation) in the pancreatic head region, and a unique peak (at 20 weeks of gestation) in the body and tail of the pancreas [30]. In the developing fetal pancreas, there is a significant presence of nerve bundles and ganglia located within the intrapancreatic mesenchyme and lobules. Thin nerve fibers can be found in the mesenchyme surrounding blood vessels and pancreatic ducts, as well as near the acini and at the edges of some pancreatic islets [29]. Furthermore, in the human fetal pancreas, nervous system structures create various complexes with endocrine cells, known as neuro-insular complexes [54]. This interaction may be influenced by neurotrophic factors such as nerve growth factor (NGF) [55] and glial cell line-derived neurotrophic factor (GDNF) [56], which are produced in epithelial progenitors [56] and differentiating endocrine cells [55] during the development of the rodent pancreas. It is now well established that all types of pancreatic cells (endocrine, exocrine, and ductal) originate from endodermal sources and develop from epithelial progenitors [7]. In contrast, neural stem cells originate from neuroectoderm and differentiate from neural crest cells that migrate to the pancreas [57]. Preganglionic cells of the sympathetic nervous system extend from the first thoracic spinal segment to the third lumbar segment, with their cell bodies located in the spinal cord, mainly in the intermediolateral gray matter [58]. The preganglionic neurons of the parasympathetic nervous system that innervate the upper body originate from cranial nerves in the brainstem, while sacral preganglionic neurons that innervate the lower body are found in the central autonomic nuclei and the sacral extension of the intermediolateral column [58]. Experimental studies on rodents and cell cultures indicate that the autonomic nervous system plays a role in regulating the proliferation and maturation of β cells and the development of islet architecture during pancreatic development [59] and influences β-cell proliferation in adult animals [60]. Neurons from the midbrain dorsal raphe nucleus and other brain regions that receive serotonin inputs (such as nucleus tractus solitarii, paraventricular hypothalamic nucleus, and arcuate nucleus) project to the pancreas via the dorsal motor nucleus of the vagus, indicating a parasympathetic pathway [61]. Additionally, neurons from the dorsal raphe and raphe pallidus, along with several regions receiving serotonergic inputs (including the paraventricular hypothalamic nucleus), project to the pancreas through the intermediolateral nucleus of the spinal cord, representing a sympathetic pathway [61].

The loss of glial cell line-derived neurotrophic factor (Gdnf) expression in mice results in fewer neurons and glial cells during the development of the pancreas, as well as a decrease in the intrinsic innervation of the islets after birth [56]. Similarly, *NGF*-deficient mice exhibit reduced sympathetic innervation of the islets [62], while mice that overexpress NGF show signs of sympathetic hyperinnervation [63]. However, the relationship between the islets and ganglionic cells, as well as the presence of hormone-secreting cells among the ganglionic cells or within nerve bundles, cannot be solely attributed to the growth of nerve fibers towards the endocrine cells. Research using NPY (neuropeptide Y) as a marker revealed a loss of sympathetic nerves in the islets of non-obese diabetic (NOD) mice that had been diabetic for just three weeks [41]. Since the activation of sympathetic nerves in the islets promotes glucagon secretion [64], this loss of islet nerves could significantly reduce a key stimulator of α cells, which is activated during hypoglycemia [65] and plays a role in the glucagon response to insulin-induced hypoinsulinemia in non-diabetic animals [65]. Nekrep et al. [66] demonstrated that *Phox2b*-null mice exhibited increased β-cell proliferation and mass without changes in overall pancreatic mass, suggesting that the effects are specific to β cells. Phox2b encodes a homeobox factor that is crucial for the specification of neural precursors derived from the neural crest.

### 3.1. Sympathetic Nerve

In the developing pancreas of mice, neural crest cells are first identified on embryonic day E10 [56]. Cells expressing the sympathetic marker VMAT2 are detected starting at E12.5 [52], and sympathetic fibers appear from E14.5 onward [59]. During the prenatal stage, both neural crest cells and sympathetic fibers are closely linked to endocrine cells [59]. However, in mice, the maturation of pancreatic islets and the establishment of sympathetic innervation, along with the development of perivascular sympathetic plexuses, take place in the early postnatal period, resembling adult structures by the third week after birth [59]. Similar findings regarding the postnatal development of sympathetic innervation in pancreatic islets have also been noted in rats [55]. In humans, the sympathetic innervation of the pancreas, including the development of perivascular and intraganglionic nerve plexuses, primarily occurs during the prenatal period, although some aspects, such as the sympathetic innervation of islet capillaries, may develop postnatally. The interactions between sympathetic terminals and endocrine cells in the fetal pancreas could be crucial for the development of the endocrine pancreas in humans [29]. The sympathetic circuit that regulates the pancreas is similar to that of the liver, gallbladder, and bile ducts. Sympathetic preganglionic neurons located in the T6-L2 intermediolateral cell column send axons through the splanchnic nerve to the celiac ganglion, where sympathetic postganglionic neurons innervate the pancreas [1].

In the pancreases of adult mice and rats, postganglionic sympathetic axons primarily innervate pancreatic blood vessels and islets [67]. Within the islets, these sympathetic axons are mainly located at the edges and interact with α cells, suggesting a direct sympathetic influence on glucagon release. In contrast, the central region of the islets sees sympathetic axons running alongside microvessels without making contact with most β cells [67]. In the exocrine tissue, sympathetic axons primarily supply capillaries, although there are occasional connections with the acini [68]. Furthermore, sympathetic axons create a network around the neuronal cell bodies in the intrapancreatic ganglia [67]. A similar pattern of sympathetic axon distribution—around blood vessels, in intrapancreatic ganglia, and at the periphery of islets—has been noted in rabbit pancreases [69]. In humans, the pancreas has less autonomic nerve innervation compared to rodents [34]. In humans, sympathetic postganglionic axons create perivascular nerve plexuses associated with acini and islets and are found surrounding the neurons in intrapancreatic ganglia [42]. The sympathetic axons in human pancreatic islets are less prevalent than in mice, and their distribution within the islets differs as well [34]. When pancreatic sections are immunostained for the sympathetic marker tyrosine hydroxylase (TH), most TH-labeled sympathetic axons in human islets are found alongside blood vessels [67] and innervate contractile cells [34]. However, sympathetic axons are also found near islet β and α cells [42].

The sympathetic nervous system is primarily responsible for promoting glucagon secretion by activating the α cell β2AR. When the sympathoadrenal system is activated, it causes the adrenal glands to release adrenaline into the bloodstream, which, along with the nervous release of noradrenaline at the islet cell level, stimulates glucagon secretion and inhibits insulin secretion. During pancreatic regeneration, βARs are functionally enhanced, leading to increased proliferation of pancreatic β cells and insulin secretion in young rats [70]. Research has shown that β cells possess more α2ARs, which inhibit insulin secretion, compared to α1ARs and β2ARs, which promote it [39]. The impact of the sympathetic nervous system on β-cell growth may involve βAR pathways. For example, a study found that a 60–70% partial pancreatectomy in rats resulted in an increase in β2ARs in the cerebrum, hypothalamus, brainstem, and pancreas, with this increase correlating with a temporary rise in DNA synthesis in the islets [70,71]. Additionally, Rodriguez-Diaz et al. reported that human islets have autonomic axons with distinct innervation patterns, where sympathetic fibers preferentially innervate the smooth muscle cells of blood vessels within the islet [34]. Sympathetic nerve endings contain noradrenaline, neuropeptide Y, and galanin. Noradrenaline binds to β2ARs on α cells, stimulating glucagon secretion, while it binds to αARs on β cells, inhibiting insulin secretion [72]. High levels of γ-aminobutyric acid (GABA), an important inhibitory neurotransmitter in the central nervous system, were found in the cytoplasm of islet β cells, with a small amount located in insulin granules [73]. Sorensen et al. identified GABAergic nerve cell bodies at the edges of pancreatic islets, with many GABA-containing processes extending into the islet mantle [73]. Saravia-Fernandez et al. observed similar structures in NOD mice, which also expressed neuropeptide Y (NPY) [74]. NPY innervation has been documented in various species, and these neuronal networks are essential for the homeostasis and metabolic regulation of pancreatic islets.

### 3.2. Parasympathetic Nerve

Preganglionic sympathetic neurons arise from the lower thoracic and upper lumbar regions of the spinal cord [75]. They leave the spinal cord via the ventral roots and connect to either the paravertebral ganglia of the sympathetic chain or the celiac and mesenteric ganglia [31]. On the other hand, parasympathetic preganglionic neurons that innervate the pancreas are located in the dorsal vagal nucleus in the brainstem. These neurons send their axons out of the central nervous system to form synapses with parasympathetic postganglionic neurons found in the pancreatic ganglia. The parasympathetic postganglionic neurons then directly innervate the pancreas [50].

Preganglionic parasympathetic neurons stimulate postganglionic neurons in the pancreatic ganglia, mainly through the activation of nicotinic acetylcholine (ACh) receptors [31]. Various neurotransmitters and neuromodulators can influence the communication between pre- and postganglionic neurons [31]. The distribution of parasympathetic neurons is broader than that of sympathetic neurons, with some areas of overlap [31]. Postganglionic neurons release multiple neurotransmitters that can either excite or inhibit, depending on the receptor type [75]. ACh is a common neurotransmitter that binds to muscarinic receptors, providing a tonic input [76]. Additionally, nitric oxide, vasoactive intestinal peptide (VIP), gastrin-releasing peptide, and pituitary adenylate cyclase-activating polypeptide (PACAP) are also released by the parasympathetic nerve [31]. Parasympathetic innervation is important for regulating pancreatic function, and its activation primarily leads to excitatory input, resulting in increased secretion from both exocrine and endocrine sources [77].

There are five types of muscarinic acetylcholine receptors (mAChRs) in the parasympathetic nervous system, labeled M1 to M5, which are found on various neurons and tissue cells in humans [78]. Notably, different islet cells exhibit different mAChR types. In the pancreatic islet β cells of the Caucasian population, M3 and M5 receptors are highly expressed [79], while islet α cells express the M2 receptor, which generates a cholinergic signal that helps regulate β-cell function through a paracrine mechanism [34]. Previous research has also shown differences in mAChR expression on human islet β cells between Caucasian and Chinese populations [80]. In Chinese populations, the M2 receptor and other cholinergic markers such as vesicular acetylcholine transporter (vAChT) and choline acetyltransferase (ChAT) are significantly present in islet β cells [80]. The M3mAChR is primarily activated during cholinergic stimulation in β cells [81]. When small animals are treated with scopolamine butylbromide early in life, the expression of M3mAChR protein in pancreatic islets decreases, highlighting the importance of muscarinic receptors in the insulin secretion of β cells. Interestingly, knockout mice that lack the M3mAChR throughout their bodies or specifically in β cells exhibit a lean phenotype, are hypophagic, hypoinsulinemic, and show poor responses of their pancreatic islets to glucose and ACh [82]. The odd-numbered mAChR subtypes function as G protein-coupled receptors that mediate the cholinergic insulinotropic response, while the even-numbered subtypes diminish the insulinotropic effects of the odd subtypes. ACh released by the vagus nerve also enhances intracellular phospholipases in β cells, leading to increased calcium release and promoting insulin vesicle exocytosis and secretion [83].

In the islets, postganglionic axons reach every type of cell in the mouse [84]. However, it was suggested that the way the parasympathetic nerves connect to the body in humans is different from how they do in mice. In humans, only a few parasympathetic axons go into the islets, and most end up in the exocrine part of the pancreas [84]. Additionally, it was shown that human β cells respond well to ACh, but α cells do not respond as much [84]. It was found that ACh, which is naturally produced in the body, helps β cells release insulin through the M3 and M5 muscarinic receptors [79]. The α cells are thought to be the main source of ACh in human islets [35]. In human islets, ACh is mostly a local signal released by α cells, unlike in rodent islets, where it mainly comes from nerves [79]. Because of this, it is hard to tell whether an effect in humans is caused by the islet cells themselves or by the nervous system.

Numerous studies have shown that the parasympathetic innervation of pancreatic β cells is crucial for regulating insulin secretion in response to glucose. For example, stimulating the vagus nerve enhances insulin release, especially when glucose levels are slightly elevated [85]. This parasympathetic activation also triggers the cephalic phase of insulin secretion, which occurs when glucose is detected in the mouth. This response can be observed in sham feeding experiments and is inhibited by vagotomy. The significance of this innervation is further highlighted by research on insulin secretion from islets transplanted into the livers of diabetic mice, which shows a marked decrease in response to oral glucose intake. The neuronal pathway involved includes direct connections from taste buds to the brainstem, which then activates the parasympathetic nerve to stimulate β cells [86]. The mass of pancreatic β cells may be influenced by the level of parasympathetic activity through the vagus nerves. It is well known that lesions in the ventromedial hypothalamus can lead to increased appetite, heightened β-cell activity, and elevated insulin levels [1]. Additionally, parasympathetic activity rises when blood glucose levels drop below normal, prompting glucagon secretion as an initial response to restore normal blood sugar levels [87].

Several studies emphasize the significance of vagus nerve efferents in the proliferation of β cells. Parasympathetic innervation of the pancreas is linked to increased β-cell proliferation and mass [61], indicating that insulin hypersecretion following hypothalamic lesions may stem from both increased β-cell proliferation and mass, as well as elevated insulin secretion [1]. In ob/ob mice, a temporary decline in β-cell proliferation was noted after a radical subdiaphragmatic vagotomy, which did not occur in control mice and was independent of food intake. This led to a reduction in β-cell mass only after several months [88]. The short-lived nature of this effect on proliferation may be due to adaptive changes in the enteric nervous system following vagal injury [89]. Additionally, changes in islet blood flow, influenced by vascular tone regulated by parasympathetic efferents [90], may also play a role in the observed effects on β-cell replication. Electrical stimulation of the vagus nerve results in a glucose-dependent increase in insulin secretion [85]. Conversely, bilateral transection of the celiac branch of the vagus nerve leads to a 50% reduction in β-cell proliferation [60]. This nervous activity may also facilitate β-cell proliferation in response to insulin resistance development in the liver [91]. As evidence mounts that specific brain regions, such as the hypothalamus and brainstem, are involved in compensatory β-cell growth and regeneration [70,92], further research is needed to clarify the roles of the central and autonomic nervous systems in maintaining and expanding pancreatic β-cell mass in response to increased physiological insulin demands. Additionally, it was reported that glucose transporter two regulates glucose-induced parasympathetic activity, which in turn stimulates β-cell proliferation [93].

The exocrine pancreas makes up most of the pancreas, and it is mostly made up of acinar cells and ductal cells [94]. Additionally, fibroblasts and pancreatic stellate cells are located near ductal cells. Acinar cells in the pancreas are responsible for making and releasing many digestive enzymes, such as amylases, lipases, and proteinases. Ductal cells help transport these enzymes to the duodenum. Centroacinar cells are a special type of duct cell found at the end of ducts, and they have been suggested as possible sources for regenerating both endocrine and exocrine parts of the pancreas. All the different cell types in the pancreas are thought to come from a common group of stem-like cells during development [94]. However, there is still debate about whether exocrine cells can change into other cell types in the adult pancreas under disease conditions [94]. When the vagus nerve fires, it can cause acinar cells in rats to grow more, and this happens mainly through a process involving cholinergic receptors [92]. More research is needed to understand this process better.

### 3.3. Other Factors

Pancreatic β cells are influenced by various external hormonal and neuronal signals, as indicated by the presence of numerous cell surface receptors [43]. These receptors include those for the gluco-incretin hormones, glucagon-like peptide-1 (GLP-1), and gastric inhibitory polypeptide (GIP), which are released by gut endocrine cells after meals, as well as for glucagon from pancreatic α cells, which can function as a paracrine signal. The receptors for these hormones are G protein-coupled receptors that activate the cAMP/protein kinase A intracellular signaling pathway, enhancing insulin secretion in response to glucose. Other neuropeptides, such as VIP, PACAP, substance P, galanin, and cholecystokinin (CCK), also stimulate glucose-induced insulin secretion through the activation of the heterotrimeric Gq protein, which regulates the IP3/Ca++ and diacylglycerol/protein kinase C pathways [24,95]. Conversely, sympathetic innervation negatively regulates β-cell activity via noradrenaline (primarily through α2-AR), galanin, and NPY receptors. These receptors are G protein-coupled and linked to the Gi protein, which decreases adenylate cyclase activity, lowers cAMP levels, and inhibits secretion [76]. Additionally, short-term increases in leptin have been shown to reduce insulin production in β cells through sympathetic activation in rodents [96]. The activation of β-cell receptors for neuropeptides found in parasympathetic nerve terminals, such as PACAP, VIP, and gastrin-releasing peptide (GRP), may also play a role in the parasympathetic regulation of β cells, although their precise physiological functions remain to be fully defined. Further research is needed to clarify the molecular mechanisms by which parasympathetic stimulation, likely through ACh/muscarinic receptors that elevate [Ca2+]i, affects β-cell growth pathways such as those regulated by extracellular signal-regulated kinase/mitogen-activated protein kinase (ERK/MAPK) and/or phosphoinositide 3-kinase/Akt signaling [60].

Mice with *glucose transporter type 2 (GLUT2)* neuronal knockout demonstrated reduced insulin secretion (in response to glucose, elevated glucagon levels, and lower β-cell proliferation and mass compared to control mice [93]). Notably, when control mice were treated with the ganglionic blocker chlorisondamine, their β-cell proliferation rate decreased by 50%. However, in *GLUT2* neuronal knockout mice, chlorisondamine did not lead to any further reduction in proliferation. This research clearly indicates that the interaction between neuronal glucose-sensing cells and islet α and β cells is essential for maintaining proper insulin and glucagon levels in the blood, a process that is impaired in type 2 diabetes.

Nerves, comprising neurons and Schwann cells, can influence the behavior of cancer cells and affect tumor development through a paracrine mechanism. Broadly, the neuroactive substances released during tumor–nerve interactions can be categorized into three primary groups: (1) neurotropic factors such as NGF, BDNF, and glial cell line-derived neurotrophic factor and others; (2) axon guidance molecules such as C-C motif chemokine ligand 2 (CCL2), C-X3-C motif chemokine ligand 1 (CX3CL1), Ephrin type-A receptor 2 (EphA2), and Slit; and (3) neurotransmitters including ACh, glutamate, glycine, adrenaline, noradrenaline, and dopamine [97]. It is not surprising that tumor cells express various receptors, including tropomyosin receptor kinase (Trk)A, TrkB, and nerve growth factor receptor (NGFR), in response to these neuroactive substances, activating downstream signaling pathways [98]. In line with this, previous research indicated that increased vagal activity due to lesions in the ventromedial hypothalamus led to enhanced proliferation of rat pancreatic β cells via a cholinergic receptor mechanism [92]. Through DNA microarray analysis of rat pancreatic islets following ventromedial hypothalamic lesions, the author found alterations in the expression of several response genes, including those related to neurotropic factors, axon guidance molecules, and neurotransmitters [99]. Currently, the author is investigating which specific factors may directly promote the proliferation of β cells.

## 4. Autonomic Nervous System and Carcinogenesis of the Pancreas

Research indicates that the autonomic nervous system may promote the development of cancer, emphasizing its involvement in the regeneration and possible malignancy of pancreatic cells. Nerves have been found to invade the tumor microenvironment, a process known as tumor neoneurogenesis or axonogenesis, and they actively contribute to cancer progression, including tumor growth and spread. In turn, tumor cells stimulate nerve growth, creating a reciprocal interaction that aids in tumor advancement [100].

The peripheral nervous system is influenced by the tissues it innervates and adapts to changes in the surrounding microenvironment [21]. Consequently, there is evidence linking neoneurogenesis in tumors to the peripheral nervous system, which includes both sympathetic and parasympathetic nerves [101]. These observations indicate that the roles of sympathetic and parasympathetic innervation differ across various cancers. Previous studies have indicated that tumor tissues are innervated and that neurotransmitters may influence tumor initiation and metastasis [102]. In several types of tumors, autonomic nerves facilitate tumor growth and metastasis by interacting with both cancer cells and the tumor microenvironment [103]. For example, in pancreatic carcinoma, intratumoral parasympathetic neurogenesis has been linked to tumor budding [6]. Although the sympathetic and parasympathetic nervous systems are traditionally seen as opposing forces, they actually work together in cancer, with sympathetic nerves promoting early tumor development and parasympathetic nerves activating later stages of metastasis [104]. To sustain their growth, tumor cells and the surrounding reactive stroma release neurotrophins to attract nerves [105]. These neurotrophins, derived from tumors, lead to the formation of axons in both autonomic and sensory nerves [106]. After the nerve recruitment phase in cancer, each type of nerve contributes to tumor growth. Sympathetic nerves facilitate the formation of new blood vessels, similar to processes in tissue development [105]. Meanwhile, parasympathetic nerves stimulate tumor cells through mitosis, initiating growth expansion [107] and providing migratory signals for metastasis [108]. Additionally, cancer cells can migrate along nerves and invade them in response to various mediators released by peripheral nerves and resident macrophages [109].

The sympathetic nervous system’s activation of βARs has been shown to enhance the growth and invasion of pancreatic cancer cells. This occurs because nerve fibers can release neurotransmitters near cancer cells, promoting their survival, proliferation, and spread [100]. Research indicates that the sympathetic neurotransmitter noradrenaline also contributes to tumor progression [110]. Specifically, noradrenaline has been found to facilitate pancreatic cancer cell invasion by influencing the expression of matrix metalloproteinases and the angiogenic cytokine vascular endothelial growth factor [111]. Elevated levels of noradrenaline may play a role in the development of various cancers [112]. Recently, Zahalka et al. [105] discovered that adrenergic nerves influence angiogenesis in the prostate cancer microenvironment by altering the metabolism of endothelial cells. They found that α2AR inhibited oxidative phosphorylation in endothelial cells, promoting angiogenesis. Hara et al. [113] demonstrated that the sympathoadrenal system regulates tumor suppressor gene functions, noting that the activation of βARs triggers a signaling pathway that leads to the degradation of p53, a product of a tumor suppressor gene in both mice and human cell lines. Tumor-associated macrophages, which are crucial components of the cancer microenvironment, significantly influence tumor development and progression [114]. These macrophages can also affect tumor cells’ resistance to cytotoxic chemotherapy by mediating the tumor microenvironment [115]. Notably, the recruitment of tumor-associated macrophages is regulated by both cholinergic and adrenergic signaling linked to the nerves. In pancreatic cancer, adrenergic signaling fosters tumor growth and decreases survival by recruiting tumor-associated macrophages, while cholinergic signaling has the opposite effect [116]. Further research indicated that vagotomy can enhance pancreatic cancer growth and shorten survival time by influencing TNFα secretion from tumor-associated macrophages [117]. Neurotransmitters and neurotrophic factors released by nerves are involved in angiogenesis by binding to receptors and encouraging the migration of endothelial cells [97]. These factors include catecholamines, ACh, dopamine, NGF, and brain-derived neurotrophic factor (BNDF) [97].

**Figure 3:**
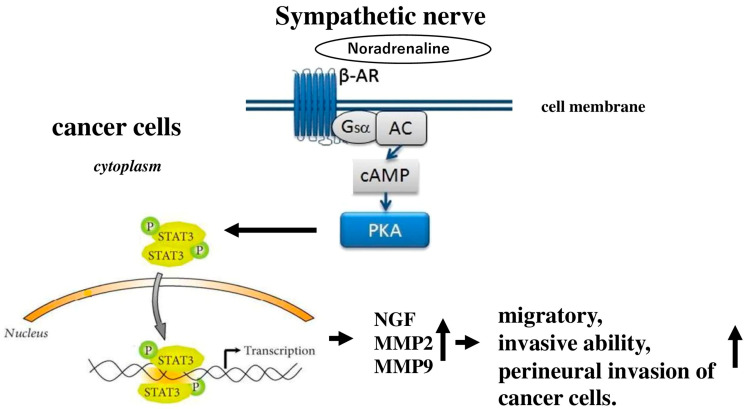
Presents a diagram showing that sympathetic nerves enhance the migratory and invasive capabilities of cancer cells, as well as their perineural invasion, by activating STAT3 via the β-AR/PKA signaling pathway. AC stands for adenylyl cyclase; PKA refers to protein kinase A; and Gsα is the G stimulatory α subunit.

Signal transducer and activator of transcription 3 (STAT3), a key transcription factor in the signal transduction and activation of transcription family, is activated through the phosphorylation of a specific tyrosine residue in response to external signals and oncogenes. It plays a significant role in various processes related to cancer progression [118]. Research has indicated that high levels of STAT3 activity are commonly observed in many human tumors, including pancreatic cancer [119]. Earlier studies have found that the sympathetic neurotransmitter noradrenaline is overexpressed in pancreatic cancer tissues [120]. A recent investigation revealed that noradrenaline activates STAT3, leading to increased production of matrix metalloproteinases in ovarian cancer cells [121]. It was discovered that noradrenaline enhances the perineural invasion of pancreatic cancer cells in a concentration-dependent manner by activating STAT3 via the βAR/protein kinase A (PKA) signaling pathway [122] (Figure 3). Inhibiting STAT3 can reduce noradrenaline-induced expression of NGF, matrix metallopeptidase (MMP) 2, and MMP 9, as well as diminish the migratory and invasive capabilities of pancreatic cancer cells. Additionally, blocking STAT3 effectively treats perineural invasion of pancreatic cancer cells in vivo.

**Figure 4:**
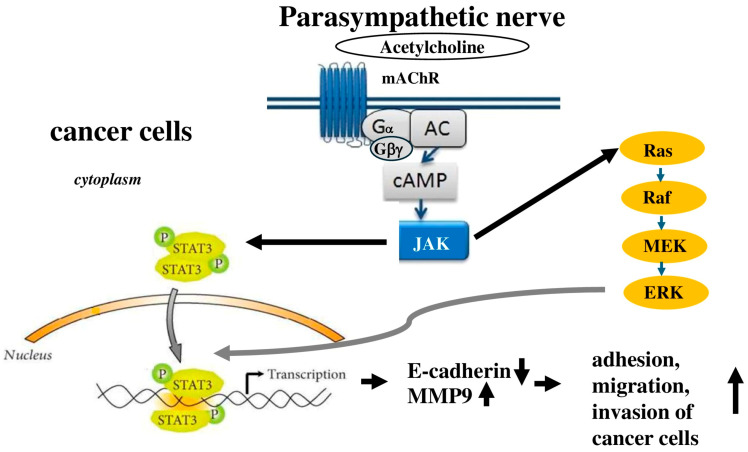
Illustrates that parasympathetic nerves facilitate the adhesion, migration, and invasion of cancer cells by activating the JAK2/STAT3 signaling cascade and the Ras/Raf/MEK/ERK1/2 pathway. AC represents adenylyl cyclase; Gα refers to the G stimulatory α subunit; and Gβγ denotes the Gβγ complex.

The vagus nerve, which consists of both parasympathetic and sensory axons, has been shown to have opposing effects in pancreatic ductal adenocarcinoma and gastric cancer. Research by Zhao et al. [123] showed that performing vagotomy or pharmacological denervation specifically on the stomach in later stages of gastric cancer reduced tumor progression and extended survival. This denervation particularly influenced the regeneration of the stem cell compartment in gastric tumors and improved the effectiveness of chemotherapy [123]. Conversely, in pancreatic cancer, the vagus nerve exhibited tumor-promoting effects, as vagotomy accelerated tumor development and growth by attracting tumor-associated macrophages and facilitating inflammation [117]. The neurotransmitter ACh acts as an autocrine growth factor in both human lung and pancreatic cancers. Song et al. [124] found that ACh promoted lung cancer cell proliferation by activating the EPK/MAPK pathways (Figure 4). Additionally, another study indicated that stimulation of the 7-nAChR receptor increased pancreatic cancer metastasis through the activation of the Janus kinase 2 (JAK2)/STAT3 signaling pathway and the Ras/Raf/MAPK/ERK kinase (MEK)/extracellular signal-regulated kinases 1 and 2 (ERK1/2) pathways [125]. Beyond promoting cell proliferation, ACh significantly enhances the adhesion, migration, and invasion of human lung cancer cells. Lin et al. [126] noted that ACh raised MMP9 expression while decreasing E-cadherin levels, both of which are linked to the migratory and invasive characteristics of lung cancer.

Recent evidence suggests that muscarinic signaling, specifically the interaction between ACh and mAChRs, plays a significant role in the progression of cancer. mAChRs are found on pancreatic stromal cells, pancreatic cancer cells, and pancreatic carcinoma. This indicates that targeting the nervous microenvironment through muscarinic signaling could be a promising strategy for regulating pancreatic cancer progression. Following this, Hayakawa et al. [127] discovered that cholinergic stimulation from nerves, via ACh release, led to increased expression of NGF in the gastric epithelium, which in turn facilitated cancer progression. Additionally, using data from the Cancer Genome Atlas, it was found that 48% of pancreatic cancer patients had alterations in genes related to neuroactive molecules or their receptors, with the most notable change being in the *neurotrophic receptor tyrosine kinase 1 (NTRK1)* gene, which encodes TrkA [128]. Further research indicated that TrkA expression was present in 1.6% of solid tumors and correlated with the number of *NTRK1* gene copies [129]. Inhibitors targeting TrkA have shown promise in treating cancers that are positive for NTRK fusions [130].

Immune checkpoint inhibitors are a type of cancer immunotherapy that boosts the immune system’s ability to identify and attack cancer cells. These therapies have shown promise in treating various cancer types [131]. Nerves, which are part of the tumor microenvironment, also interact with the immune system, potentially aiding tumor progression through inflammation [132]. These intricate systems engage with each other on multiple levels. Neuroendocrine and neuronal pathways play a role in regulating immune responses, as many of their molecular signals and receptors belong to the same superfamily. Research indicates that the autonomic nervous system can directly influence the immune system.

## 5. Autonomic Nervous System and Apoptosis of the Pancreas

**Figure 5:**
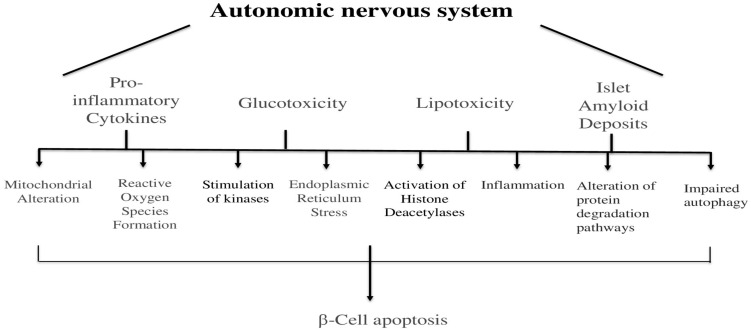
Illustrates the autonomic nervous system and apoptosis of the pancreas.

The autonomic nervous system also plays a role in causing cell death in the pancreas. The death of beta cells happens because of several reasons, such as harmful inflammation chemicals, high sugar levels, fat buildup, and protein clumps in the islets [133] (Figure 5). These factors can also lead to cell death by changing how mitochondria work, creating harmful chemicals, activating certain enzymes, stressing the cell’s internal structure, turning on certain proteins, causing inflammation, changing how proteins are broken down, and stopping the body’s natural cleaning process [133]. However, how these factors connect with the autonomic nervous system is not completely understood yet.

The cannabinoid 1 receptor is crucial for the autonomic nervous system as it regulates neurotransmission and affects various physiological functions. This G-protein-coupled receptor is mainly located in the central and peripheral nervous systems, including autonomic pathways [134]. Cannabinoid 1 receptors are predominantly found in presynaptic and axonal areas, limiting their activity to synaptic sites. Endocannabinoids are produced when cells depolarize, and they primarily act on β cells through the cannabinoid 1 receptor present on all β cells, influencing both basal and glucose- and incretin-stimulated insulin secretion, as well as β-cell reactions to different stressors. Evidence suggests that the cannabinoid 1 receptor negatively impacts β-cell mass regulation. This aligns with findings that disrupting the cannabinoid 1 receptor genetically or pharmacologically enhances insulin receptor signaling through insulin receptor substrate (IRS)2/AKT in β cells, resulting in increased β-cell mass. Furthermore, cannabinoid 1 receptors influence the expression of the anti-apoptotic protein Bcl-2 and the cell cycle regulator cyclin D2 in pancreatic β cells. When β-cell lines were treated with the synthetic cannabinoid receptor agonist WIN55,212-2, there was a reduction in Bcl-2 and cyclin D2 expression, leading to cell cycle arrest in the G0/G1 phase and caspase-3-dependent apoptosis. Conversely, the genetic deletion and pharmacological inhibition of cannabinoid 1 receptors in injured mice resulted in elevated levels of Bcl-2 and cyclin D2 in pancreatic β cells [135]. Additionally, cannabinoid 1 receptor activation in isolated human and rodent macrophages triggered the NOD-like receptor family pyrin domain containing 3-apoptosis-associated speck-like protein containing a CARD (NLRP3-ASC) inflammasome, which increased the production of the macrophage chemotactic protein (MCP)-1 and the release of cytokines such as IL-1β and IL-18, ultimately causing β-cell apoptosis [136].

Furthermore, a partial deficiency in Pdx-1 leads to increased β-cell apoptosis, resulting in reduced β-cell mass and diabetes in both rodents and humans [137]. A recent study also found that glucotoxicity and lipotoxicity contribute to β-cell death and dysfunction due to oxidative stress-induced reductions in Pdx-1 expression [137].

## 6. Autonomic Nervous System and Non-Apoptotic Cell Deaths of the Pancreas

Table 1 illustrates the influence of the autonomic nervous system on non-apoptotic mechanisms. In endocrine cells, pyroptosis is primarily recognized as a novel form of programmed cell death mediated by pro-inflammatory processes [138]. This type of cell death is characterized by gasdermin-induced pore formation in the cell membrane, cell swelling, rapid lysis, and the release of pro-inflammatory cytokines such as interleukin-1β and interleukin-18. Extensive research has demonstrated that pyroptosis typically occurs through activation of the caspase-1-dependent canonical pathway as well as the caspase-4/5/11-dependent non-canonical pathway. Autophagy is an evolutionarily conserved process of degradation and recycling that is triggered by factors such as viral infections, endoplasmic reticulum stress, oxidative stress, and nutrient deprivation [139]. It is a lysosome-dependent pathway responsible for recycling intracellular components and removing damaged organelles, generally regarded as a survival mechanism in response to stress [138]. All forms of autophagy culminate in lysosomal degradation. Moreover, global deletion of the T1D-risk gene *Ctsh*, which encodes a lysosomal cysteine protease, impairs insulin secretion and increases fasting blood glucose levels, highlighting the critical role of autophagy in maintaining β-cell homeostasis [140].

In exocrine cells, necroptosis—a form of programmed inflammatory cell death—plays a role in the development of acute pancreatitis [141]. Necroptosis is a caspase-independent, inflammatory cell death pathway initiated when immune ligands such as tumor necrosis factor (TNF) bind to their receptors on the cell surface. This triggers receptor-interacting protein kinase 1 (RIPK1) to associate with RIPK3 in the cytoplasm, forming a complex known as the necrosome. The necrosome then signals to mixed lineage kinase domain-like protein (MLKL), which moves to the plasma membrane and induces necroptosis. This process causes membrane permeabilization and leakage of cytoplasmic contents, releasing danger-associated molecular patterns (DAMPs) that act as alarm signals to provoke inflammation, activate the immune response, and initiate tissue repair [142]. Ferroptosis is another type of programmed cell death characterized by the buildup of lipid peroxides within cells. It is marked by iron accumulation, which generates reactive oxygen species and leads to the destabilization of cell membranes [143]. Unlike other cell death forms, such as apoptosis, ferroptosis can be inhibited by specific agents such as ferrostatin-1. Solute carrier family 7 member 11 (SLC7A11), functioning as an antiporter, is widely recognized for its role in suppressing ferroptosis [143].

These processes frequently appear in acute pancreatitis, diabetes, and pancreatic cancer, occasionally initiated by oxidative stress, cytokine storms, or metabolic overload. Excessive sympathetic activation during stress can worsen necroptosis or ferroptosis through catecholamine-induced oxidative stress. In contrast, parasympathetic signaling might offer protection by encouraging autophagy and reducing inflammatory cell death. Additionally, fibers of the autonomic nervous system interact with immune cells in the pancreas, affecting cytokine patterns that promote pyroptosis. Nevertheless, the exact relationship between these factors and the autonomic nervous system remains unclear.

## 7. Autonomic Nervous System and Metabolism of the Pancreas

**Figure 6:**
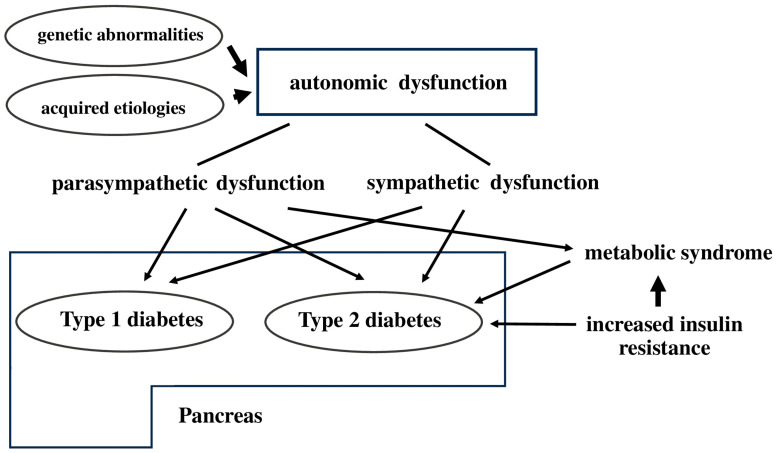
Illustrates the autonomic nervous system and metabolism of the pancreas.

Autonomic neuropathy is a long-term problem that often comes with diabetes mellitus. It can happen before someone develops type 2 diabetes or metabolic syndrome. Both genetic and non-genetic causes can lead to autonomic issues. If the parasympathetic or sympathetic nervous system is not working properly, it might cause type 2 diabetes by lowering how much insulin the body makes, or it might lead to metabolic syndrome by making the body more resistant to insulin. Insulin resistance is a major factor in the development of metabolic syndrome [144] (Figure 6).

Pancreatic islets receive direct signals from the autonomic nervous system, which is part of the central nervous system [1]. This suggests that the autonomic nervous system could be another way through which ghrelin influences glucose regulation. There is some evidence indicating that ghrelin signaling through the vagus nerve plays a role in insulin release. It is also possible that ghrelin may inhibit insulin secretion through indirect pathways, such as activating visceral afferent nerves in the hepatic portal vein [145] or through sympathetic responses [146]. Glucose sensors located in the hepatic portal vein and hypothalamic neurons also play a role in insulin secretion in response to food intake and hypoglycemia [147]. Importantly, these are also areas where ghrelin may exert its effects [148]. This information suggests that, in addition to its direct actions, ghrelin might also reduce insulin secretion indirectly through neural pathways. For instance, ghrelin infusion into the portal vein inhibited glucose-stimulated insulin secretion, but this effect was not observed when infused into the femoral vein, and hepatic vagotomy or intra-portal atropine reduced this inhibitory effect [149]. In humans, administering ghrelin increased plasma adrenaline [146], reduced heart rate variability [150], and lowered both heart rate and blood pressure [150], indicating that ghrelin may trigger sympathetic and/or parasympathetic responses that could influence islet secretion. Supporting this idea, an intact vagus nerve is necessary for many of ghrelin’s physiological effects in both mice and humans [150], yet no studies have directly investigated whether ghrelin regulates insulin secretion through the autonomic nervous system. In humans with vagotomy, exogenous ghrelin similarly had no impact on autonomic parameters or food intake [150]. In rats, ghrelin inhibited glucose-stimulated insulin secretion when infused into the portal vein but not the femoral vein, and this effect was diminished by hepatic vagotomy [147].

In addition to regulating circadian rhythms of many physiological functions, melatonin is involved in regulating autonomic nervous function and blood pressure [151]. It has been suggested that melatonin impacts glucose metabolism by affecting both beta-cell insulin secretion and insulin sensitivity [152]. It is well described that pancreatic alpha and beta cells express melatonin receptors MT1 and MT2 [152], and evidence shows that melatonin action on the pancreatic melatonin receptors modulates insulin secretion and glucagon release in a diurnal fashion [153]. More research is needed to understand how melatonin influences glucose regulation and whether there is a causal relationship between melatonin concentrations and glucose tolerance.

In rats, two insulin-coding genes, *insulin 1 (Ins1)* and *insulin 2 (Ins2)*, are located on chromosome 1 [154]. *Ins1* originated from an *Ins2* transcript and was integrated into the genome through an RNA-mediated duplication-transposition event, as indicated by certain structural feature analyses. The PI3K/AKT pathway is a critical intracellular signaling cascade activated by insulin, playing key roles in glucose uptake, inhibition of gluconeogenesis, and promotion of cell survival and growth. Pdx-1 (pancreatic and duodenal homeobox 1) is a transcription factor essential for pancreatic β-cell development and function. It directly regulates insulin gene transcription. The phosphoinositide 3-kinase (PI3K)/AKT pathway positively regulates Pdx-1, enhancing its activity and nuclear localization. I noted that overexpressing the Pdx-1 gene enhances *Ins1* mRNA expression but does not affect *Ins2* mRNA levels in an insulinoma cell line, suggesting that the levels of rat insulin 1 and insulin 2 peptides may vary under certain conditions [154].

Phosphatase and tensin homolog deleted on chromosome 10 (PTEN) serves as a significant negative regulator of the mechanistic target of rapamycin (mTOR) and PI3K/Akt pathways [155]. When the *PTEN* tumor suppressor gene is inactive, often due to deletion, it leads to the activation of the PI3K/Akt/mTOR pathway [155]. It has been reported that PTEN enhances insulin signaling by increasing *Ins2* mRNA levels. Additionally, PTEN has been found to be secreted in exosomes and released into the extracellular space. The author also proposed that overexpressing PTEN could lead to increased expression of the *Ins2* gene, which is one of the phosphorylated genes that counteract *Ins2* through the insulin/insulin-like growth factor (IGF)-1 receptor [155].

## 8. Conclusions

The human pancreas consists of approximately 1 million islets, and for these islets to secrete hormones in pulses, their oscillatory activity must be well coordinated or synchronized. If the oscillations of individual islets were not coordinated, they could cancel each other out, leading to a loss of pulsatile secretion. While there has been some advancement in understanding how individual islet oscillations are generated, the processes that facilitate communication and synchronization between islets are still not well understood. Although it has been proposed that a neural network in vivo connects the activities of various islets into a cohesive unit [156], there is limited direct experimental evidence to back this idea, and the specific characteristics and components of this network remain unclear. The autonomic nerves in the islets play an important role in helping the body release insulin during the early stage of eating, in making sure the islets work together as a group so they can release hormones in a rhythmic way, and in helping the islets release hormones more efficiently when the body is under stress, such as when blood sugar is too low [25]. The amount and timing of insulin release can change based on many factors, including glucose levels [157]. The timing changes are how the islets respond to changes in glucose. When many islets are exposed to the same glucose signal, they can start to work together and release insulin at the same time. This suggests that timing changes in insulin release are important for keeping blood sugar levels balanced and for helping the islets work coordinately [157]. Additionally, using computer models, scientists think that signals from the nervous system, specifically cholinergic signals, might help control the natural rhythm of insulin release in the body [158]. Furthermore, the exocrine function of the pancreas is essential for the digestive system, as it releases digestive enzymes and bicarbonate ions into the duodenum to facilitate food digestion. This intricate process is regulated by a network of hormonal and autonomic nervous system signals that control enzyme secretion in response to food intake [159]. The interaction between the pancreas’s endocrine and exocrine functions involves complex signaling mechanisms, including the insulin-glucagon feedback loop, which is vital for managing both glucose metabolism and the secretion of exocrine enzymes [160].

Table 2 shows the functional comparison regarding the autonomic innervation of the pancreas. These advancements regarding regulation of insulin secretion, pancreatic regeneration, apoptosis and carcinogenesis, and gene expression and growth factors provide a deeper understanding of how the autonomic nervous system interacts with the pancreas, offering potential avenues for therapeutic interventions in pancreatic diseases.

## Figures and Tables

**Table 1:** Autonomic nervous system influence on non-apoptotic cell deaths.

Type of Cell Death	Features	Relevance to Pancreas
**Endocrine cells**		
Pyroptosis	Caspase-1 or 4/5/11 mediated, inflammatory	Linked to islet inflammation in diabetes
Autophagic cell death	Excessive autophagy leading to cell demise	Observed in stress-induced β-cell dysfunction
**Exocrine cells**		
Necroptosis	Regulated necrosis via RIPK1/RIPK3/MLKL	Seen in pancreatitis
Ferroptosis	Iron-dependent lipid peroxidation	Implicated in acinar cell damage

**Table 2:** Autonomic innervation of the pancreas: functional comparison.

Function/Target Area	Sympathetic Nervous System	Parasympathetic Nervous System
**Endocrine secretion (islets)**	Inhibits insulin release (via noradrenaline on α2AR)	Stimulates insulin release (via vagal ACh signaling)
**Exocrine secretion (acinar cells)**	Inhibitory (via vasoconstriction and reduced perfusion)	Stimulatory (via ACh acting on muscarinic receptors)
**Pancreatic blood flow**	Vasoconstriction (reduced perfusion)	Vasoconstriction (reduced perfusion)
**Neurotransmitters involved**	Noradrenaline, NPY, GABA, Galanin	Ach, Nitric oxide, VIP, GRP, PACAP
**Receptor types**	Adrenergic receptors (α, β), TRPV1 channels	Muscarinic acetylcholine receptors (M1–M5)
**Impact on glucose homeostasis**	Promotes hyperglycemia (via insulin suppression, glucagon stimulation)	Promotes normoglycemia (via insulin stimulation)
**Cell regeneration or proliferation**	Stimulates β cells (via noradrenaline on β2AR)	Stimulates β cells and acinar cells (via vagal ACh signaling)

## Data Availability

No new data were generated.

## Abbreviations

ACh: Acetylcholine; AR: Adrenergic receptor; bHLH: Basic helix-loop-helix; BNDF: Brain-derived neurotrophic factor; CCK: Cholecystokinin; CCL2: C-C motif chemokine ligand 2; CX3CL1: C-X3-C motif chemokine ligand 1; Dnmt1: DNA (cytosine-5)-methyltransferase 1; EphA2: Ephrin type-A receptor 2; ERK1/2: Extracellular signal-regulated kinases 1 and 2; ERK/MAPK extracellular signal-regulated kinase/Mitogen-activated protein kinase; FoxA1: Forkhead box A1; FoxA2: Forkhead box A2; GABA: γ-aminobutyric acid; Gdnf: Glial cell line-derived neurotrophic factor; CHOP: C/EBP Homologous Protein; DAMPs: danger-associated molecular patterns; GIP: Gastric inhibitory polypeptide; GRP: Gastrin-releasing peptide; GLP-1: Glucagon-like peptide-1; GLUT2: Glucose transporter type 2; Hex: Hematopoietically expressed homeobox; Hnf6; Hepatocyte nuclear factor 6; IGF: Insulin-like growth factor; IRS: Insulin receptor substrate; Ins: Insulin; Isl1: Islet1; JAK2: Janus kinase 2; MEK: MAPK/ERK kinase; MLKL: Mixed lineage kinase domain-like protein; MMP: Matrix metallopeptidase; mTOR: mechanistic target of rapamycin; NeuroD1: Neurogenic differentiation 1; Ndrg4: N-myc downstream-regulated gene 4: Ngn3: Neurogenin 3; Nkx6.1: NK homeobox 6.1; Nkx6.2: NK homeobox 6.2; NGF: Nerve growth factor; NGFR: Nerve growth factor receptor; NOD: Non-obese diabetic; NPY: Neuropeptide Y; NTRK1: Neurotrophic receptor tyrosine kinase 1; NLRP3-ASC: NOD-like receptor family pyrin domain containing 3-Apoptosis-associated speck-like protein containing a CARD; PACAP: Pituitary adenylate cyclase-activating polypeptide; Pax4: Paired box gene 4; Pax6: Paired box gene 6; Pdx1: Pancreatic and duodenal homeobox 1; PI3K: Phosphoinositide 3-kinase; PKA: Protein kinase A; Prox1: Prospero homeobox 1; PTEN: Phosphatase and tensin homolog deleted on chromosome 10; Ptf1a: Pancreas-specific transcription factor 1a; Rfx: Regulatory factor X; RIPK1: Receptor-interacting protein kinase 1; Sox17: Sree-box transcription factor 17; STAT3: Signal transducer and activator of transcription 3; SLC7A11: Solute carrier family 7 member 11; TGFβ: Transforming growth factor beta; TRPV1: Transient receptor potential cation channel subfamily V member 1; Trk: Tropomyosin receptor kinase; VIP: Vasoactive intestinal peptide.
